# Role of RNA-Binding Proteins in MAPK Signal Transduction Pathway

**DOI:** 10.1155/2011/109746

**Published:** 2011-04-05

**Authors:** Reiko Sugiura, Ryosuke Satoh, Shunji Ishiwata, Nanae Umeda, Ayako Kita

**Affiliations:** ^1^Laboratory of Molecular Pharmacogenomics, School of Pharmaceutical Sciences, Kinki University, 3-4-1 Kowakae, Higashi-Osaka 577-8502, Japan; ^2^Research Fellow of the Japan Society for the Promotion of Science, 1-8 Chiyoda-ku, Tokyo 102-8472, Japan

## Abstract

Mitogen-activated protein kinases (MAPKs), which are found in all eukaryotes, are signal transducing enzymes playing a central role in diverse biological processes, such as cell proliferation, sexual differentiation, and apoptosis. The MAPK signaling pathway plays a key role in the regulation of gene expression through the phosphorylation of transcription factors. Recent studies have identified several RNA-binding proteins (RBPs) as regulators of MAPK signaling because these RBPs bind to the mRNAs encoding the components of the MAPK pathway and regulate the stability of their transcripts. Moreover, RBPs also serve as targets of MAPKs because MAPK phosphorylate and regulate the ability of RBPs to bind and stabilize target mRNAs, thus controlling various cellular functions. In this review, we present evidence for the significance of the MAPK signaling in the regulation of RBPs and their target mRNAs, which provides additional information about the regulatory mechanism underlying gene expression. We further present evidence for the clinical importance of the posttranscriptional regulation of mRNA stability and its implications for drug discovery.

## 1. Introduction

Cells need to respond to a variety of signals including extracellular stimuli, environmental stresses, and developmental signals as well as intrinsic information, to properly regulate gene expression and thereby execute biological functions. Given the multiplicity and complexity of the regulation of gene expression, failure to coordinate these regulatory mechanisms can contribute to the onset and progression of diseases such as cancer. Gene transcription and its regulatory mechanisms, together with recent genome-wide approaches for gene transcription analysis, have attracted the most attention with regard to gene regulation. However, it has become clear that additional stages in the gene expression cascade, including posttranscriptional events such as the control of mRNA degradation, stability, location, and translation, are equally crucial and require sophisticated regulation by various intracellular signaling pathways [1]. RNA-binding proteins have been shown to control the expression of numerous proteins by binding to the respective mRNA species encoding proto-oncogenes, growth factors, cytokines, transcription factors, and other proteins in various cell types [2]. In particular, the phosphoregulation of RNA-binding proteins by MAPKs and/or kinases downstream of MAPKs, which can control the degradation or translation of target mRNAs, has attracted increasing attention. One of the most well-studied model systems for the study of MAPK regulation is the fission yeast *Schizosaccharomyces pombe* (*S. pombe*). *S. pombe*, with its powerful genetics, is an excellent model system for the study of the mechanisms of signaling pathways in higher eukaryotes. In *S. pombe*, homologs of many signaling molecules involved in tumorigenesis, such as Ras, Rho, Protein kinase C, and MAPK, as well as drug targets, including TOR (target of Rapamycin) and the phosphatase calcineurin (target of the immunosuppressant drug FK506), are highly conserved. Spc1/Sty1/Phh1 and Pmk1 in *S. pombe* are homologs of mammalian p38 and ERK, respectively. The purpose of this review is to highlight the significance of several data using the fission yeast model system as well as other studies in higher eukaryotes that uncovered the cross-talk mechanism between MAPKs and RNA-binding proteins (RBPs) in the regulation of posttranscriptional gene expression, biological phenomena, and signal transduction [3].

## 2. RNA-Binding Proteins as Regulators of MAPK Signaling

**Figure 1:**
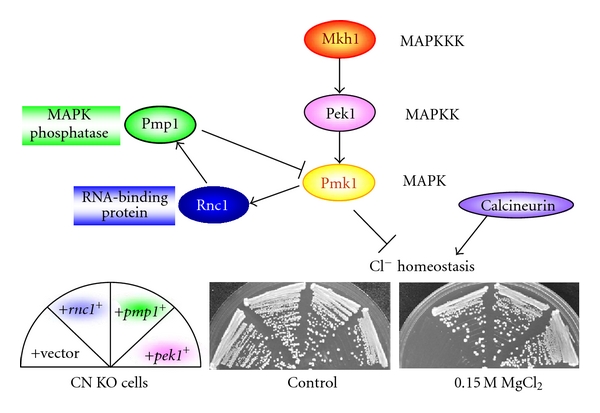
Molecular genetic approach to identify regulators of MAPK signaling using fission yeast model system. Upper panel: Calcineurin phosphatase and Pmk1 MAPK pathway play antagonistic roles in Cl^−^ homeostasis of fission yeast. Molecular genetic approach utilizing this genetic interaction identified regulators of Pmk1 MAPK. Lower panel: Calcineurin deletion cells (CN KO) failed to grow in the presence of 0.15 M MgCl_2_. Overexpression of multicopy suppressor genes, including *pmp1^+^* (MAPK phosphatase), *pek1^+^* (MAPK kinase), and *rnc1^+^* (KH-type RNA-binding protein) suppressed the Cl^−^ sensitivity of the calcineurin deletion cells.

Sugiura et al. developed a genetic approach to identify regulators of MAPK signaling on the basis of the antagonistic relationship between calcineurin phosphatase and MAPK Pmk1 in the regulation of Cl^−^ homeostasis in fission yeast (Figure 1) [4]. By utilizing this genetic interaction, Sugiura et al. identified several negative regulators of MAPK signaling, including *pmp1^+^*, *pek1^+^*, and *rnc1^+^* (Figure 1). Pmk1 MAPK is a homologue of ERK/MAPK in mammals and regulates cell morphology, cytokinesis, ion sensitivity, and cell integrity in fission yeast [5]. The first gene identified encodes a dual-specificity MAPK phosphatase Pmp1 that dephosphorylates Pmk1 MAPK, thereby inhibiting Pmk1 signaling (Figure 1) [4]. The second gene identified was *pek1^+^*, encoding an MAPK kinase (MAPKK), which phosphorylates and activates Pmk1 in its phosphorylated form, thus acting as an upstream MAPKK for Pmk1 (Figure 1) [6]. Surprisingly, Pek1 in its unphosphorylated state binds phosphorylated Pmk1, thereby inhibiting Pmk1 MAPK signaling. Hence, Pek1 acts as a phosphorylation-dependent molecular switch. The third identified gene encodes a novel KH-type RBP (Figure 1), which shares significant amino acid similarity with human Nova-2, human FMR-1, and *Caenorhabditis elegans* GLD-1. The third gene was designated as *rnc1^+^* (RNA-binding protein that suppresses calcineurin deletion 1) because overexpression of the *rnc1^+^*gene suppressed Cl^−^ hypersensitivity of calcineurin-knockout cells (Figure 1). 

### 2.1. MAPK Phosphatase mRNA as a Target of RNA-Binding Proteins

**Figure 2:**
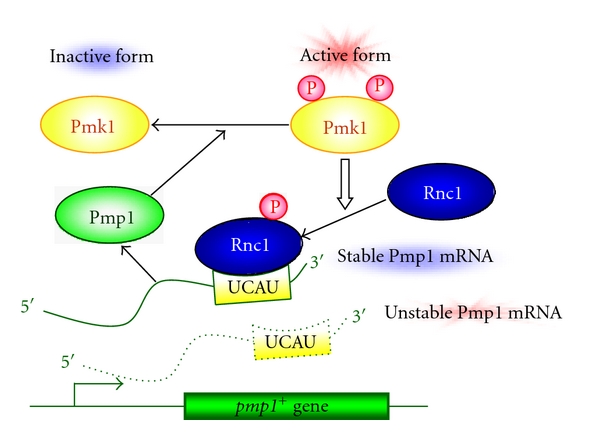
Negative feedback regulation of MAPK signaling mediated by an RNA-binding protein Rnc1. Rnc1 binds to the UCAU repeats located in the 3′-UTR of the Pmp1 mRNA. The activated Pmk1 phosphorylates Rnc1, leading to the enhanced activity of Rnc1, thereby binding to and stabilizing an otherwise unstable Pmp1 mRNA. Upregulated Pmp1 dephosphorylates and inactivates Pmk1, thus creating a negative-feedback loop that regulates the MAPK signalling.

Rnc1 turned out to be a negative regulator of Pmk1 MAPK since Pmk1 MAPK was heavily phosphorylated in Rnc1-knockout cells but was barely phosphorylated in cells overproducing Rnc1. Based on the hypothesis that Rnc1 affects the metabolism of specific cellular mRNAs involved in the regulation of the Pmk1 signaling, the effects of Rnc1 on the mRNA stability of the MAPK phosphatase Pmp1 were examined, because Pmp1 was identified in the same genetic screen. The hypothesis is correct since Rnc1 binds and stabilizes an otherwise unstable Pmp1 mRNA, thus providing a novel functional link between MAPK signaling and an RBP through the mRNA stabilization of MAPK phosphatase (Figure 2) [7]. These findings are particularly important since Rnc1 directly affects the expression of MAPK phosphatase, thereby controlling the strength and duration of MAPK signaling pathway. 

Pmp1 is highly similar to members of the dual-specificity MAPK phosphatase MKP-1 (MAPK phosphatase or DUSP1; dual-specificity phosphatase1), which plays a critical role in the negative regulation of MAPK signaling by dephosphorylating and inactivating ERK MAPK, JNK, and p38 [8–12]. Notably, Rnc1 recognizes and binds to the UCAU repeats in the 3′-UTR (untranslated region) of the Pmp1 mRNA (Figure 2) [7]. Mutations in the UCAU sequences greatly destabilize Pmp1 mRNA owing to the lack of binding of Rnc1 to Pmp1 mRNA [7]. Interestingly, the Nova-1 KH-type RNA-binding protein, which has been implicated in the pathogenesis of paraneoplastic opsoclonus-myoclonus ataxia (POMA), a disorder associated with breast cancer and motor dysfunction, binds to an element containing repeats of the tetranucleotide UCAU [8]. It would be intriguing to speculate that the mRNA stability of human MAPK phosphatase might also be regulated by an RNA-binding protein with or without KH-motifs. 

Cell exposure to cytokines, growth factors, cell stresses, or activated oncogenes leads to the activation of MAPK and to the upregulation of mRNAs encoding a subset of MAPK phosphatase genes [9–18]. This induction of MAPK phosphatase expression has been extensively documented, and it is considered to involve transcriptional induction by various transcription factors, such as CREB and AP-1, via both MAPK-dependent and MAPK-independent pathways [19]. However, as shown in the study of Pmp1 in fission yeast, MAPK phosphatase gene expression is regulated not only by its transcription rate but also by its rate of mRNA degradation in higher eukaryotes. Wong et al. reported that heat shock increases MKP-1 gene expression at both the transcriptional and posttranscriptional levels and the latter involves increased mRNA stability of MKP-1 [20]. Moreover, It has been shown that DUSP1/MKP-1 mRNA is stabilized by oxidative stress-induced binding of RBPs HuR and NF90 [21, 22]. In addition, HuR promotes MKP-1 mRNA recruitment to translation machinery and its translation efficiency. 

Recently, Lin et al., Emmons et al., and Bros et al. suggested the importance of TTP, a zinc finger-AU-rich elements (AREs) RBP, in the regulation of MKP-1 mRNA [23–25]. TTP has been shown to bind to AREs located in the 3′-UTR of unstable mRNAs and to direct its target mRNAs for degradation. By utilizing RIP-Chip analysis in human dendritic cells, Emmons et al. identified 393 messages as putative TTP targets, 33 of which contained AREs, including dual specificity phosphatase1 (DUSP1/MKP1) mRNA. They further confirmed that the wild-type TTP significantly inhibits DUSP1/MKP1 expression by utilizing luciferase-DUSP 3′-UTR reporter, which contains repeats of the AUUUA sequences. Bros et al. revealed the role of TTP in the degradation of MKP-1 mRNA. They showed elevated expression of TTP target mRNAs, including MKP-1, in TTP^−/−^ dendritic cells. Lin et al. also showed that biotinylated MKP-1 AREs could bring down HuR and TTP in preadipocytes. Therefore, MKP1 mRNA is a target of several RBPs including HuR and TTP, and these RBPs are involved in the modulation of MKP-levels and MAPK activity through mRNA (de)stabilization of MAPK phosphatase, thus indicating that MKP-1 expression is tightly regulated transcriptionally and posttranscriptionally in many organisms, which ensures a rigid feedback control of MAPK signaling.

### 2.2. Feedback Regulation of MAPK Signaling by an RNA-Binding Protein

Interestingly, Rnc1 protein in fission yeast is regulated by MAPK phosphorylation. There are six putative MAPK-phosphorylation sites in Rnc1 protein and Pmk1 MAPK directly phosphorylates Rnc1 at T50 *in vivo* and *in vitro *[7]. Notably, this phosphorylation by MAPK regulates the RNA-binding activity of Rnc1, that is, the unphoshorylatable T50A mutant Rnc1 failed to bind Pmp1 mRNA while the phospho-mimic T50E mutation enhanced the RNA-binding activity of Rnc1 [7]. Therefore, when Pmk1 signaling is activated, Pmk1 MAPK phosphorylates Rnc1 and the phosphorylated Rnc1 protein more potently binds and stabilizes Pmp1 mRNA, resulting in upregulation of Pmp1 MAPK phosphatase, followed by the inactivation of Pmk1 signaling (Figure 2) [7]. Thus, the identification of Rnc1 as a target of the MAPK signaling cascade has revealed a novel feedback mechanism for the posttranscriptional regulation of MAPK phosphatase gene expression [7]. 

Similar negative-feedback mechanisms of the MAPK signaling pathway through posttranscriptional mRNA stabilization of MAPK phosphatase have been reported in other organisms. As described above, Casals-Casas et al. reported posttranscriptional mRNA stabilization of MKP-1 in macrophages and showed that this stabilization mechanism was partly dependent on p38 MAPK [19]. Recently, Bermudez et al. described the posttranscriptional regulation of DUSP6/MKP-3 phosphatase by MERK/ERK signaling and hypoxia [26]. They showed that the MEK/ERK pathway stabilizes *dusp6* mRNA levels via its 3′-UTR. Moreover, hypoxia, a hallmark of tumor growth, increases *dusp6* mRNA stability in a MEK activity-dependent manner. Interestingly, 2 RNA-binding proteins, TTP, and PUM2, a homolog of *Drosophila pumilio* protein, destabilize *dup6* mRNA via its 3′-UTR. Therefore, although there is a diversity of the MAPK pathways concerned between *S. pombe *and higher eukaryotes, these studies suggest a conserved mechanism of feedback-regulation mechanism of MAPK signaling through the mRNA stabilization of MAPK phosphatase. Thus, it is intriguing to speculate that some RBPs responsible for the mRNA stability of the MAPK phosphatase gene(s) might be regulated by p38 or ERK MAPK in higher eukaryotes. Attractive candidates for such RBPs may include TTP, since the well-known link between MK2 and TTP provides a plausible explanation for posttranscriptional regulation of MKP-1 by the p38 MAPK pathway.

## 3. RNA-Binding Proteins as Targets of MAPK Regulation

The MAPK pathway is known to play an important role in regulating gene expression, and this regulation has been primarily attributed to the activation of transcription factors via MAPK-dependent phosphorylation. However, since the time p38 MAPK was found to play a major role in the regulation of mRNA stability [27], accumulating evidence suggests that the stability of mRNAs can be controlled by RBPs under the regulation of various MAPK signaling pathways in different cellular systems [28–31]. These include tristetraprolin (TTP) [32–34], nucleolin [35], hnRNP-K [36, 37], hnRNPA0 [38], HuD [39], and the poly(A)-binding protein PABP1 [40], as well as Rnc1, Nrd1, and Csx1 in fission yeast [7, 41, 42].

**Figure 3:**
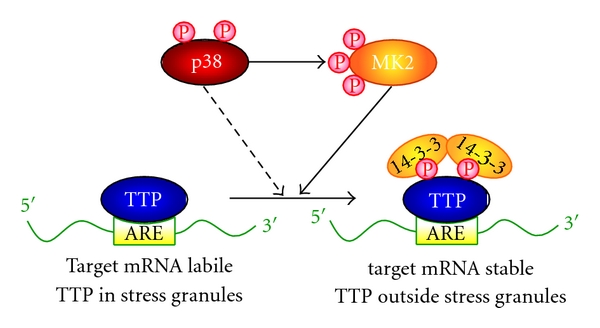
Control of ARE-containing mRNA degradation by TTP. Unphosphorylated TTP binds to the ARE and promotes degradation of the mRNAs. MK2 phosphorylates TTP at two serine residues, and this phosphorylation allows binding of 14-3-3 adaptor proteins and stabilization of the ARE-containing target mRNAs, such as TNF*α*. TTP can also be phosphorylated by p38. Phopshorylation also excludes TTP from stress granules.

Since the activation of p38 MAPK and its downstream MAPK-activated protein kinase-2 (MAPKAPK-2 or MK2) signaling pathway leads to the stabilization of AU-rich elements (AREs)-containing transcripts including various inflammation genes, ARE-binding proteins are likely targets for the p38 MAPK/MK2 pathway [43]. One of the well-characterized RBPs is TTP, which acts as a substrate of p38 MAPK/MK2. TTP binds to the ARE motifs in the 3′-UTR of several cytokine mRNAs including TNF*α* mRNA and targets them for degradation, thereby suppressing inflammation [33]. TTP^−/−^ mice developed severe inflammatory symptoms, including cachexia, spontaneous arthritis, dermatitis, and neutrophilia, mainly because of the overproduction of TNF*α* resulting from the increased stability of the TNF mRNA and subsequent higher rates of secretion of the cytokine [44]. Notably, macrophages derived from TTP^−/−^ MK2^−/−^ double knockout mice showed highly increased amounts of TNF almost identical to those in TTP-deficient mice, whereas in MK2-null mice, the TNF level was dramatically reduced in MK2-deficient animals compared to the wild type [45]. This genetic evidence strongly suggests that TTP is the main downstream element of MK2 in the posttranscriptional regulation of TNF *in vivo*. MK2 directly phosphorylates TTP at 2 serine residues (Ser52 and Ser178 in mouse TTP) *in vitro* and *in vivo* (Figure 3) [46]. Furthermore, TTP can be directly phosphorylated by p38 (Figure 3) [32]. 

Notably, phosphorylation by MK2 inhibits TTP activity. Several studies have described the molecular mechanisms underlying this regulation, although this subject is controversial. The phosphorylation of TTP by MK2 facilitates the dissociation of TTP from the ARE motif, thereby allowing the biosynthesis of TNF*α* [45]. Previous studies have also shown that MK2 is required for the maintenance of normal TTP levels and phosphorylation by MK2 leads to stabilization of TTP and inhibition of its mRNA destabilization activity. Notably, the phosphorylation of TTP at 2 serine residues (Ser52 and Ser178) allows binding of 14-3-3 adaptor proteins (Figure 3) [47, 48]. Furthermore, Stoecklin et al. showed that MK2-dependent phosphorylation of TTP releases it from stress granules and inhibits degradation of mRNAs containing AREs (Figure 3) [48]. Interestingly, recent studies by Marchese et al. and Clement et al. described a molecular mechanism for the regulation of TTP function by MK2, involving the inhibition of recruitment of a deadenylase complex [49, 50]. In addition to TTP, MK2 also phosphorylates poly(A)-binding protein [40] and hnRNPA0 [51], thereby regulating their mRNA-binding capacity. Moreover, p38 MAPK phosphorylates the KH-type splicing regulatory protein (KSRP) and inhibits its binding and destabilization of mRNAs coding for proteins involved in the differentiation of myoblasts into myotubes, thus indicating that p38 regulates mRNA turnover by targeting KSRP [52]. 

Interestingly, the budding yeast p38 homolog Hog1 was shown to be essential for stabilization of these ARE-containing mRNAs [53], thus indicating that MAPKs also regulate mRNA stability in lower eukaryotes. In fission yeast, there is an interesting association between p38 and Csx1, an RBP crucial for survival following oxidative stress in *S. pombe *[42]. Csx1 is an RRM-type RBP with significant amino acid similarity to TIA1 in higher eukaryotes. Csx1 is phosphorylated upon oxidative stress in a Sty1-dependent manner. Csx1 associates with and stabilizes *atf1^+^* mRNA, which encodes a member of the ATF/CRE-binding protein family of transcription factors, including ATF2, in higher eukaryotes [42]. Moreover, Csx1 controls expression of the majority of the genes regulated by Spc1 p38 and Atf1 in response to oxidative stress. Notably, HuR RBP in higher eukaryotes stabilizes ATF-2 mRNA by binding to the 3′-UTR of ATF-2 mRNA in response to polyamine concentrations [54]. Consistently, HuR overexpression and depletion modulates ATF-2 mRNA abundance and resistance to apoptosis. These studies reveal a highly conserved mechanism of controlling MAPK-regulated transcription factors as well as several physiological responses, including oxidative stress and apoptosis through RBP.

## 4. Other Instances of MAPK-Mediated Phosphoregulation of RBPs: Cell Cycle Control Mediated by Modulation of mRNA Stability

Recent paper by Satoh et al. revealed an interesting link between RBP and cell cycle control mediated by modulating myosin mRNA stability [41]. They identified Nrd1, a highly conserved RNA-recognition motif (RRM)-type RBP, as a regulator of Cdc4, an essential myosin light chain, a key regulator of cytokinesis in fission yeast [55, 56]. Nrd1 binds to Cdc4 mRNA *in vivo* and *in vitro*, thereby stabilizing Cdc4 mRNA and regulating cytokinesis in fission yeast. Nrd1 shows significant sequence similarity with TIA-1 and the TIA-1-related (TIAR) proteins in higher eukaryotes [57, 58]. It has been reported that both TIA-1 and TIAR bind to RNA, with the preferred binding sequence being U-rich including the UCUU motifs present in the mRNAs [59, 60]. Notably, the mutation in the two UCUU sequences (*cdc4*M1M2) within the Cdc4 ORF abrogated the binding between Nrd1 and Cdc4 mRNA, and the *cdc4*M1M2 mutant cells exhibited cell separation defects, suggesting that the loss of Nrd1 binding affects cytokinesis.

Interestingly, Nrd1 is also phosphorylated by Pmk1 MAPK at two threonine residues (T40 and T126) and this phosphorylation negatively regulates Nrd1 activity to bind and stabilize Cdc4 mRNA and cytokinesis. Nrd1^DD^, the phosphorylation mimic version of Nrd1, only weakly binds to Cdc4 mRNA, while Nrd1^AA^, the unphosphorylatable version of Nrd1, in which T40 and T126 are substituted by alanine residues, strongly binds to Cdc4 mRNA [41]. The deletion of Pmk1 MAPK, similar to Nrd1 overexpression, stabilizes Cdc4 mRNA, thus providing the first example of MAPK-mediated cell cycle control through mRNA stabilization of myosin via RBP. 

There are also interesting examples of cell-cycle control by modulation of mRNA stability in higher eukaryotes, especially with regard to DNA damage checkpoints [61]. Reinhardt et al. reported the involvement of MK2 activity in the regulation of Gadd45*α* mRNA stability [38] and showed that the p38/MK2-dependent phosphorylation of 3 key targets involved in RNA regulation-hnRNP0, TIAR, and PARN-stabilizes an otherwise unstable Gadd45*α* mRNA through its 3′-UTR, resulting in increased Gadd45*α* protein levels. Gadd45*α* itself is then required to maintain MK2 activity, which is critical for prolonged Cdc25 inhibition and maintenance of a G_2_ arrest [38]. Thus, the positive feedback loop involving p38/MK2/Gadd45*α* is essential to allow cells to recover from DNA damage, before committing to the next mitotic cell division. These reports on RBP-mediated cell-cycle control through mRNA stability provide further evidence for the phosphoregulation of RBPs through MAPK, thereby revealing an additional level of cell cycle regulation.

## 5. Clinical Importance of the Posttranscriptional Regulation of mRNA Stability and Its Implications for Drug Discovery

Considerable number of genes that code for AU-rich mRNAs, including cytokines, chemokines, growth factors, RBPs, and transcription factors, are commonly involved in both chronic inflammation and cancer [62]. Therefore, stabilization of the ARE-mRNAs via RBP can cause prolonged responses that subsequently may lead to chronic inflammatory diseases, such as rheumatoid arthritis and cancer. Indeed, neutralizing antibodies to TNF*α*, one of the most important proinflammatory cytokine containing AREs, prevent arthritis in transgenic mice expressing ARE-lacking stable TNF*α* transcripts, which produce pathological overexpression of TNF*α* [63, 64]. Thus, abrogation of posttranscriptional pathways that mediate the expression of these proinflammatory genes may serve as a clinical target of inflammation. Furthermore, since the expression of MAPK phosphatase is closely linked to inflammation as well as malignancies of various cancer cells, targeting the mRNA expression of MKPs could represent a promising therapeutic target and elucidation of these mechanisms would be highly important for understanding the mechanism of cancer and inflammation. Since abnormal activation of the Ras/Raf/MAPK pathway can lead to cancer and since the p38/MK2 pathway plays a crucial role in inflammation, these MAPKs, together with the RBPs that regulate MAPKs, are attractive targets for new therapies for both cancer and/or inflammatory diseases such as Crohn's disease or rheumatoid arthritis. A large body of evidence from preclinical studies indicate a crucial role of p38 MAPK signaling in inflammation, and drugs that target p38 or MK2 and those that inhibit the development of inflammatory arthritis in mouse models are being investigated for the treatment of patients with rheumatoid arthritis and inflammatory bowel disease [65–67].

## 6. Concluding Remarks

**Figure 4:**
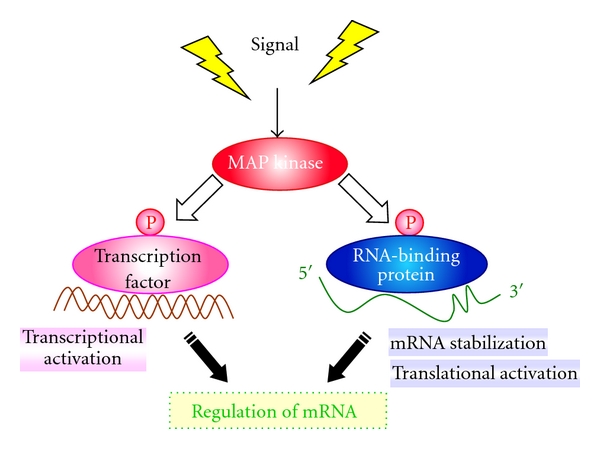
The regulation of mRNA in MAPK signaling. MAP kinase signaling pathways regulate both transcriptional and posttranscriptional gene expression in various organisms. MAPK-signaling mediated phosphorylation regulates transcription factors and/or RNA-binding proteins, which resulted in mRNA regulation. Precise regulatory mechanism(s) of these proteins by MAPK signaling are not fully understood.

We have presented several examples of functional relationships and cross talk regulatory mechanisms between RBPs and MAPK signaling in fission yeast model system and have drawn some parallels with animal cells. A recent paper by Belle et al. on the global investigation of protein half-lives in budding yeast indicates that the protein products of genes that are coregulated on the transcriptional level also tend to be coregulated on the level of protein turnover [68]. Similarly, it would be intriguing to see the correlation between transcriptional and posttranscriptional regulation, since ERK MAPKs and the p38/MK2 axis regulate both the transcriptional and posttranscriptional gene expression of various immediate early genes involved in inflammation and stress responses [69]. Further studies are required to clarify the complex interplay between these transcriptional and posttranscriptional events during inflammation and cancer, and its regulation by RBPs and various signaling pathways including MAPKs pathways and others (Figure 4). Moreover, it would be important to elucidate the basis for the cooperative action of p38 MAPK and ERK pathways for optimal ARE-mRNA stabilization [70]. To clarify these mechanisms, the use of a simple eukaryotic model organism such as fission yeast could contribute to the field, since many RBPs and signaling pathways, including MAPKs and the major pathways of mRNA degradation and transcription, are highly conserved. Altogether, these data highlight an emerging role for RBPs as regulators of MAPK signaling and future targets for drug discovery.
